# Testing the effectiveness of a motivational interviewing-based brief intervention for substance use as an adjunct to usual care in community-based AIDS service organizations: study protocol for a multisite randomized controlled trial

**DOI:** 10.1186/s13722-017-0095-8

**Published:** 2017-11-17

**Authors:** Bryan R. Garner, Heather J. Gotham, Stephen J. Tueller, Elizabeth L. Ball, David Kaiser, Patricia Stilen, Kathryn Speck, Denna Vandersloot, Traci R. Rieckmann, Michael Chaple, Erika G. Martin, Steve Martino

**Affiliations:** 1 RTI International, P. O. Box 12194, 3040 E. Cornwallis Rd., Research Triangle Park, NC 27709-2194 USA; 2 School of Nursing and Health Studies, University of Missouri-Kansas City, 2464 Charlotte St, Kansas City, MO 64108 USA; 3 University of Nebraska Public Policy Center, 215 Centennial Mall South, Suite 401, Lincoln, NE 68588 USA; 4 Vandersloot Training and Consulting, 11845 NW Stone Mt. Lane, #108, Portland, OR 97229 USA; 5 Public Health and Preventive Medicine, Oregon Health and Science University, 3181 SW Sam Jackson Park Rd. CB669, Portland, OR 97239 USA; 6 National Development and Research Institutes, Inc, 71 West 23rd Street, New York, NY 10010 USA; 7 Department of Public Administration and Policy, Rockefeller College of Public Affairs and Policy, University at Albany, 1400 Washington Avenue, Milne 300E, Albany, NY 12222 USA; 8 Rockefeller Institute of Government, State University of New York, 1400 Washington Avenue, Milne 300E, Albany, NY 12222 USA; 9 Department of Psychiatry, VA Connecticut Healthcare System, Yale University, 950 Campbell Avenue (116B), West Haven, CT 06516 USA

**Keywords:** Substance use, HIV, AIDS, type 2 hybrid trial

## Abstract

**Background:**

In 2010, the first comprehensive National HIV/AIDS Strategy for the United States was released and included three goals: (1) reducing the number of people who become infected with HIV, (2) increasing access to care and improving health outcomes for people living with HIV, and (3) reducing HIV-related health disparities and health inequities. In 2013, as part of its effort to help address the National HIV/AIDS Strategy, the National Institute on Drug Abuse (NIDA) funded a type 2 effectiveness-implementation hybrid trial titled the Substance Abuse Treatment to HIV Care (SAT2HIV) Project. Aim 1 of the SAT2HIV Project tests the effectiveness of a motivational interviewing-based brief intervention (MIBI) for substance use as an adjunct to usual care within AIDS Service Organizations (ASOs) as part of its MIBI Experiment. Aim 2 of the SAT2HIV Project tests the effectiveness of implementation and sustainment facilitation (ISF) as an adjunct to the Addiction Technology Transfer Center (ATTC) model for training staff in motivational interviewing as part of its ISF Experiment. The current paper describes the study protocol for the ISF Experiment.

**Methods:**

As part of a multisite randomized controlled trial, individuals with comorbid HIV/AIDS and problematic substance use are randomized to receive either the ASOs’ usual care (control condition) or usual care plus a MIBI for substance use (experimental condition) delivered by trained ASO case-management staff. Primary outcome measures are reductions in days of primary substance use, number of substance-related problems, times engaging in risky behaviors, days of non-adherence to HIV medications, and increases in substance use treatment. As part of this paper, we describe the trial protocol in accordance with the Standard Protocol Items: Recommendations for Interventional Trials guidelines.

**Discussion:**

If successfully able to implement MIBI as an effective adjunct to usual care, the current trial may have a significant impact on increasing the capacity of ASOs to address problematic substance use among individuals living with HIV/AIDS. Reducing the prevalence of problematic substance use among individuals living with HIV/AIDS within the United States may lead to significant improvements on key performance measures (i.e., the HIV Care Continuum and the 90-90-90 target).

*Trial registration* ClinicalTrials.gov: NCT02495402

**Electronic supplementary material:**

The online version of this article (10.1186/s13722-017-0095-8) contains supplementary material, which is available to authorized users.

## Background

### Background and rationale

In 2010, the first comprehensive National HIV/AIDS Strategy for the United States was released and included three primary goals [1]. The first goal was reducing the number of people who become infected with HIV. This goal is paramount given estimates that there are approximately 50,000 new HIV infections each year within the United States [2] and that lifetime treatment costs of each new HIV infection are approximately $400,000 (in 2015 dollars) [3], suggesting the overall lifetime treatment costs for HIV in the United States increases by approximately $20 billion a year. The second goal was increasing access to care and improving health outcomes for people living with HIV. This goal is important given estimates that 60% of the 1.2 million Americans infected with HIV are not engaged in HIV care and 63% are not prescribed antiretroviral therapy (ART) [4], when ART can significantly reduce the risk of developing AIDS [5] and new HIV infections [1, 6]. The third goal was reducing HIV-related health disparities and health inequities, which are significant in the United States [7]. For example, the overall rate of HIV infection for Blacks is eight times the overall rate for [7], and approximately 75% of HIV/AIDS cases are among men [8]. Furthermore, a gender by race disparity exists; the HIV rate for Black men is seven times the rate for White men, and the HIV rate for Black women is 19 times the rate for White women [7].

**Fig. 1:**
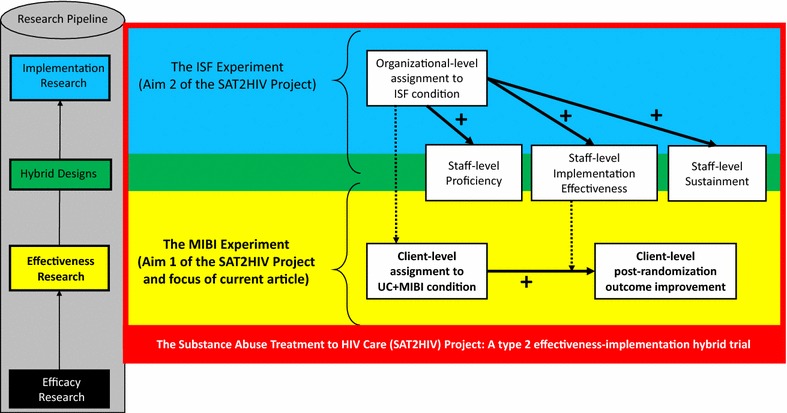
Conceptual overview of the MIBI experiment within the context of the parent SAT2HIV Project. Note: MIBI = Motivational Interviewing-based Brief Intervention; ISF = Implementation and Sustainment Facilitation; bolded arrows represent hypothesized relationships. Dashed arrows represent interactions and cross-level interactions that will be examined

In 2013, as part of its effort to help bolster the National HIV/AIDS Strategy, the National Institute on Drug Abuse (NIDA) released a multipronged expansion of HIV- and AIDS-related research that included a request for research on the integration of substance use services within HIV/AIDS settings [9]. In 2014, NIDA awarded funding for two 5-year projects. One, titled “Implementation to Motivate Physician Response to Opioid Dependence in HIV Settings,” proposed a stepped wedge design to test the effectiveness of a multifaceted implementation strategy in terms of increasing implementation of naloxone and buprenorphine/naloxone within HIV primary care organizations [10]. The second, titled “Substance Abuse Treatment to HIV Care” (SAT2HIV), proposed a type 2 effectiveness-implementation hybrid trial design [11, 12]. As shown in Fig. 1, Aim 1 of the SAT2HIV Project tests the effectiveness of a motivational interviewing-based brief intervention (MIBI) for substance use as an adjunct to usual care within AIDS Service Organizations (ASOs) as part of its multisite MIBI Experiment. Aim 2 of the SAT2HIV Project tests the effectiveness of implementation and sustainment facilitation (ISF) as an adjunct to the Addiction Technology Transfer Center’s (ATTC) model for training staff in motivational interviewing as part of its ISF Experiment. The current paper describes the study protocol for the MIBI Experiment, which has been written in accordance with the SPIRIT guidelines [13, 14] (see Additional file 1). The study protocol for the ISF Experiment, also written in accordance with the SPIRT guidelines, has been prepared separately [15]. With this background, we describe below the objective, design, and methods for the SAT2HIV Project’s MIBI Experiment.

### Overview of study objective and design

The SAT2HIV Project’s MIBI Experiment was conceptualized in 2013, when research on brief interventions for drug use was regarded as needed [16] and promising [17–19]. The primary objective of the MIBI Experiment was to test the effectiveness of MIBI as an adjunct to UC for substance use within ASOs (i.e., Aim 1 of the SAT2HIV Project). Consistent with the extant research [17, 20], we hypothesized that relative to the UC condition, the MIBI condition would result in significant reductions in primary substance use, substance-related problems, engagement in risky behaviors, as well as in significant increases in receipt of substance use treatment, and ART medication adherence. In terms of design, the MIBI Experiment is a multisite randomized controlled two-group (UC vs. UC + MIBI) effectiveness trial whose primary endpoint of interest is primary substance use during the 4-weeks following randomization. Randomization used a 1:1 allocation ratio. An effectiveness trial was selected because our primary interest was whether MIBI would work when used within the real-world conditions of ASOs, which is a design that more directly informs those making decisions about appropriate services to implement in practice settings [21]. Since the SAT2HIV Project was funded, research failing to support brief interventions for drug use within primary care settings has been reported [22, 23], as has research supporting brief interventions for drug use within HIV primary care settings [24]. Building upon the extant research evidence base, the SAT2HIV Project’s MIBI Experiment will help advance research on brief interventions for drug use, especially in HIV service settings. Consistent with Aharonovich and colleagues’ explanation for their positive findings relative to the null findings of others [22, 23], we believe the potential for reducing substance use (alcohol and other drug use) may be greater in HIV service settings than in general primary care. To the extent that this is true, addressing substance use within HIV service settings has the potential to have several important public health impacts, including improvements in HIV quality of care [25, 26], medication adherence [27–30], and viral suppression [31–33].

## Methods

### Participants, interventions, and outcomes

#### Study setting

The MIBI Experiment is being conducted in community-based ASOs (targeted N = 39) located across the United States. ASOs conduct HIV prevention efforts and provide medical case management services (e.g., retention in care, medication adherence, referral to social services and specialty treatment) to individuals living with HIV/AIDS, including support services for their families and friends. ASOs are distinct from HIV primary care organizations, which as defined by the Centers for Disease Control and Prevention, provide medical services including prescriptions for ART, CD4 T-lymphocyte testing, and/or HIV viral load testing [34].

#### Eligibility criteria

Consistent with the intent of effectiveness trials [21], there were limited eligibility criteria beyond the clinical indication of interest (i.e., comorbid HIV/AIDS and substance use disorder). Specifically, the eligibility criteria for the study were: (1) living with HIV/AIDS, (2) being 18+ years of age, and (3) acknowledging use of at least one substance within the past 28 days and endorsing two or more substance use disorder symptoms during the past 12 months. The only study exclusion criterion was not being able to speak English.

#### Interventions

*Usual care.* ASOs rarely systematically screen for or assess substance use as part of their UC process. It is even rarer for ASOs to have adequately trained staff to provide substance use services. Consequently, when individuals with comorbid HIV/AIDS and problematic substance use are identified, many ASOs are only able to offer these individuals a referral to a local substance use treatment organization. Thus, for the current experiment, UC consisted of referral to formal addiction treatment, mutual-help services, or both.

**Fig. 2:**
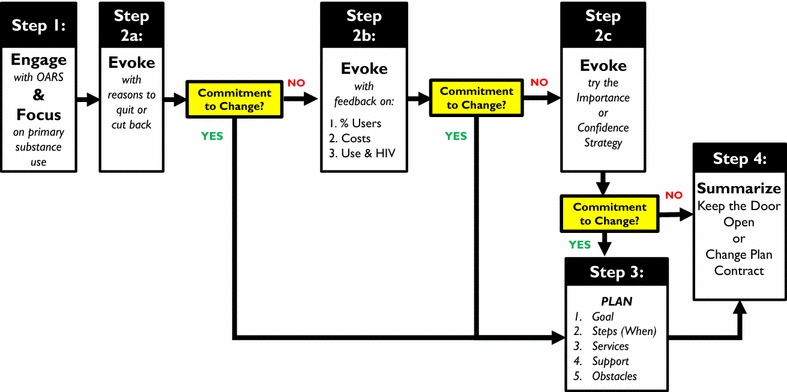
Visual illustration of the multi-step motivational interviewing-based brief intervention (MIBI)

*Motivational interviewing*-*based brief intervention.* In addition to UC, all eligible and consenting client participants randomized to the UC + MIBI condition receive the project’s MIBI. MIBI is a single-session 20- to 30-min MIBI delivered by one of the ASO’s two trained case-management staff, which we refer to hereafter as BI Staff. The intervention uses a step-by-step format (see Fig. 2).

- Step 1 aims to have BI Staff engage clients and help them focus on a primary substance (i.e., one that caused them the most problems) and its relation to HIV. BI Staff try to understand clients’ motivation for stopping substance use and receiving HIV care.
- Step 2 aims to build upon Step 1 by strategically evoking clients’ reasons for change (called change talk). Step 2 provides up to three opportunities (Steps 2a–2c) for motivational enhancement. In Step 2a, BI Staff summarize clients’ arguments for and against change and then have the clients identify additional reasons from a checklist for quitting or cutting down substance use. BI Staff ask clients to elaborate on identified reasons and then ask a key question to determine their substance use goal. If clients commit to quitting or cutting back on substance use, BI Staff proceed to Change Planning (Step 3). If clients remain ambivalent about changing substance use, BI Staff move to Step 2b. In Step 2b, BI Staff offer clients personalized feedback about how their use compares with that of others, the annual cost of their use, and how substance use typically affects Highly Active Antiretroviral Therapy (HAART) adherence, morbidity, and mortality among people infected with HIV. BI Staff summarize and emphasize change talk. If clients remain noncommittal, BI Staff use additional motivational enhancement strategies (e.g., importance or confidence ruler technique) to evoke motivation for change (Step 2c).
- Step 3 involves developing a change plan to strengthen clients’ commitment to cut back or quit substance use. BI Staff cover the following elements: steps clients might take, identification of when each step will be used, review of available substance use treatment services, and identification of social supports and obstacles.
- Step 4 summarizes the MIBI session. For clients who committed to change and developed a change plan, BI Staff use a contract with clients to summarize the change plan and fortify the clients’ commitment to it. For clients who remain unsure about changing their substance use, BI Staff seek to “keep the door open” by thanking clients for talking about their substance use and encouraging them to discuss it again at their next visit.

Training BI Staff in MIBI includes well-established methods: (1) an ATTC-developed online course on motivational interviewing (1 h per week for 5 weeks; *A Tour of Motivational Interviewing* at www.healtheknowledge.org); (2) an ATTC-run skill-based training workshop (2 days); and (3) ongoing ATTC-led performance review, feedback, and coaching based on ratings of audio recorded MIBI sessions with practice clients. All members of the training team are members of the Motivational Interviewing Network of Trainers [35]. Before implementing MIBI with actual study participants, each BI Staff is required to demonstrate MIBI proficiency (i.e., a score of 4+ [out of 7] on half or more of 10 adherence ratings and on half or more of 10 competence ratings) with at least one practice client. All practice MIBIs are rated by one of the ATTC trainers, with MIBI integrity measured in accordance with the Independent Tape Rater Scale [36].

#### Outcomes

Adapted from the Addiction Severity Index, 5th Edition, [37] primary outcome measures included: days of primary substance use, number of substance-related problems, times engaging in risky behaviors, days of substance use treatment, and ART medication adherence (see Table 1). To be consistent with our team’s other research testing the MIBI protocol [38], the specific time point of interest was 28 days post-randomization, the method of aggregation for each condition was the group-centered mean, and the participant-level analysis metric was the participant’s final value adjusted for the participant’s baseline value. Additionally, several secondary outcomes (e.g., urgency to change, intentions to change, commitment to change, self-efficacy to change) are measured and examined as mechanisms of change for the MIBI [12].

**Table 1:** Instruments, instrument-related procedures, and primary outcome measures

Instruments (time; compensation)	Collection time-points and procedures			Primary outcome measures
	Enrollment (pre-baseline)	Baseline (t = 0 weeks)	Follow-up (t = 4 weeks)	
Substance Use Screener (1–5 min; $0)	X^a,b^			
Project Introduction Script (1–2 min; $0)	X^a,b^			
Informed Consent (5–10 min; $0)	X^a,b^			
Assurance of Consent (1–2 min; $0)	X^a,b^			
Locator Form (5–10 min; $0)		X^b^		
Baseline Assessment (20–40 min; $20)		X^b^		Baseline measurement of each primary outcome was completed as part of the baseline assessment, which was adapted from the Addiction Severity Index, 5th Edition. [37] Descriptions of the primary outcome measures are provided below
Follow-up Assessment (20–40 min; $20)			X^c^	*Days of primary substance use* A continuous measure (ranges from 0 to28) of the number of days participants used their primary substance during the past 28 days *Number of substance-related problems* A continuous measure (ranges from 0 to 11) of the number of substance use disorder symptoms participants had during the past 28 days *Times engaging in risky behaviors* A continuous measure (no specified range) of the number of times participants engaged in unprotected sex, injection drug use, or needle sharing during the past 28 days *Days of substance use treatment* A continuous measure (ranges from 0 to 28) of the number of days participants attended residential treatment, outpatient treatment, or self-help group meetings during the past 28 days *Antiretroviral therapy (ART) medication adherence* A continuous measure (ranges from 0 to 28) of the number of days participants missed at least one dose of their HIV medications during the past 28 days

^a^Screening and recruitment staff trained to complete; ^b^ brief intervention (BI) staff trained to complete; ^c^ condition blinded research staff trained to complete

#### Participant timeline

**Fig. 3:**
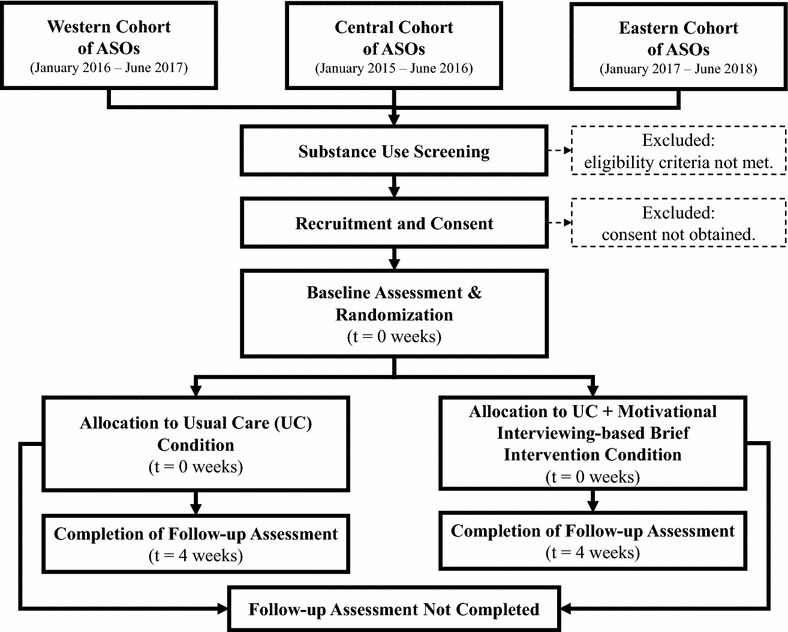
Flow of participants

Figure 3 depicts the standardized participant flow diagram used by each of three cohorts of ASOs. A brief (1–5 min) substance use screener is utilized by trained staff (including but not limited to BI Staff) to identify eligible client participants (see eligibility criteria section). Immediately following the completion of the substance use screener, staff read a standardized project introduction and ask potential participants if they are interested in learning more. Individuals with an expressed interest are then read the informed consent and given a copy. Within a week of completing the screening and written informed consent, one of the organization’s two BI Staff members administers the project’s 30-minute baseline assessment and participant locator form, schedules a 4-week follow-up assessment appointment, and randomizes (see allocation section) the participant to one of the two study conditions. Immediately following randomization, the BI Staff administers the organization’s usual care protocol (e.g., referral to local substance treatment organization) and, when applicable, the MIBI session. Participation concludes with the completion of a 30-minute, 4-week post-randomization follow-up assessment.

#### Sample size

Thirty-nine participating ASOs, each with 48 participants and an expected intraclass correlation coefficient of .05, would provide 80% power to detect a statistically significant (*p* < .05) difference for effect sizes of .20 or greater [39]. In anticipation of organizations recruiting 75% of their target number of participants and in anticipation of an 80% follow-up completion rate, each organization targets 72 client participants. Thus, the sample size is anticipated to be between 1872 (39 × 48 = 1872) and 2592 participants (39 × 72 = 2808).

#### Recruitment

As part of the participant recruitment process, each trained ASO staff person utilizes a standardized participant recruitment packet that includes (1) a substance use screener, (2) a project introduction sheet, (3) the informed-consent form, and (4) the assurance of consent. In addition to having ASOs incorporate the participant screening and recruitment process into their regular workflow (e.g., new client intakes, client reevaluations), ASOs also placed copies of the project’s standardized research project flyer on their bulletin boards and the bulletin boards of other locally relevant organizations (e.g., department of public health). Each ASO seeks to recruit 12 client participants per month over a 6-month recruitment period, and ASOs receive $50 per participant randomized. Additionally, starting in month 4 of the recruitment period, individual BI Staff earn $20 for their fourth participant randomized each month, $30 for their fifth participant, and $50 for their sixth participant. To maintain staff awareness of the extent to which they are successfully achieving their monthly and overall participant recruitment goals, during the first week of each month, one of the project’s coordinators generates and distributes to each ASO a customized end-of-month report. This report details the ASOs’ prior-month and cumulative screening and recruitment progress.

### Assignment of interventions

#### Allocation

Immediately after completing the baseline assessment and locator form, client participants are randomized to one of the project’s two conditions. A blocked randomization sequence (blocking size of 6) generated via a blocked randomization program [40] determines condition assignment. Within each of the 39 ASOs, each of the two trained BI Staff has a lock box containing 36 sequentially numbered tamper-evident security envelopes. Within each envelope is a randomization slip indicating the condition assignment. The randomization envelope is opened in front of the participant. Upon completion of the randomization process, BI Staff update a secure centralized recruitment tracking log, which a project coordinator monitors multiple times per week.

#### Blinding (masking)

The BI Staff and the client participants are not blinded to study condition. The follow-up assessment staff, who are RTI International employees, are blinded to study condition.

### Data collection, management, and analysis

#### Data collection methods

*Training for data collection staff.* Beyond successfully completing RTI’s online human subject protection training [41], project staff must successfully complete all applicable project trainings before assisting with data collection. For staff administering the instruments during screening and recruitment (approximately 2–5 staff per ASO), the 1-hour one-on-one training process consists of (1) confirming that the trainee fully read the training manual; (2) answering questions about the training manual; (3) demonstrating how to administer the screening and use recruitment-related instruments (see Table 1); (4) having the trainees practice the screening and using recruitment-related instruments with the trainer following a standardized answer script; and (5) reviewing the guidelines for storing, transmitting, and destroying data. Upon completion of this training, trainees receive a $25 gift card. For staff assisting with completion of the baseline assessment and locator form and participant randomization (the two BI staff at the ASO), the 1–hour one-on-one training process consists of (1) confirming that the trainee fully read the training manual; (2) answering questions about the training manual; (3) providing a standardized overview of the baseline assessment, locator form, and participant randomization process; (4) having the trainees practice the initial interview, locator form, and randomization process with the trainer following a standardized answer script; and (5) reviewing the guidelines for storing, transmitting, and destroying data. Upon completion of this training, trainees receive a $25 gift card. For staff administering the follow-up assessment (approximately 5 staff employed and compensated by RTI), a 2-hour group training process consists of (1) reviewing the project goals and design, (2) providing a standardized overview of the follow-up assessment, (3) practicing the follow-up assessment as a group with the trainer following a standardized answer script, (4) reviewing the procedures for contacting and confirming the identify of project participants, and (5) reviewing the participant compensation process.

*Instruments and instrument*-*related procedures for data collection.* Table 1 lists the project’s instruments (e.g., substance use screener, informed consent, baseline assessment, locator form, follow-up assessment) and instrument-related procedures (e.g., participant time, participant compensation, administration staff, collection points).

#### Data management

*Data management guidelines and procedures for ASOs.* To ensure appropriate data management at each ASO, the following data storage, transmission, and destruction guidelines were created and approved by the governing Institutional Review Board (IRB): (1) hard copies of completed project documents will be temporarily stored in a secure location only accessible to project staff, (2) electronic copies of completed project documents and files will be stored in a secure electronic folder or on an encrypted thumb drive that is only accessible to project staff and only long enough to allow the documents to be transmitted to RTI staff, (3) electronic copies of project documents will be transmitted to RTI staff only through RTI’s encrypted SharePoint website, (4) audio recordings of brief interventions will not contain any participant-identifying information, (5) audio recordings of brief interventions will be transmitted to RTI staff only through RTI’s secure website, (6) electronic copies of project documents and files will be destroyed immediately after being transmitted to RTI staff, (7) hard copies of project documents will be shredded after RTI has confirmed receipt of the electronic copies, and (8) all applicable Health Insurance Portability and Accountability Act procedures and guidelines [42] will be adhered to. RTI research staff helped each BI Staff develop a data storage, transmission, and destruction protocol, which had to be subsequently reviewed and approved by each ASO’s designated safety person before the BI Staff was allowed to assist with the project.

*Data quality assurance procedures.* Upon receipt of electronic copies of project documents, a member of the research staff at RTI reviews them. In addition to working with the respective ASO staff to resolve issues in real-time, each ASO receives a monthly data quality feedback report that lists data quality issues identified during the previous calendar month and any necessary corrective actions.

*Data entry procedures.* After the data quality assurance procedures have been completed, RTI staff enter all study document data into a Voxco-based [43] data entry program that resides on RTI’s enhanced security network. Although quality assurance checks are built into the data entry program, 10% of entered study documents are randomly selected for additional quality assurance checks by a data entry supervisor.

*Data storage procedures.* All data from the research project are stored on RTI’s enhanced security network, which adheres to the security standards of the Federal Information Processing Standards (FIPS) “moderate” level of security categorization [44], implementing multiple security measures, including two-factor authentication.

#### Statistical methods

An intention-to-treat analysis approach (i.e., all participants analyzed as randomized) will be used. Though missing data is anticipated to be minimal (i.e., less than 5%), hot-deck imputation [45, 46] will be used. All analyses will be conducted with HLM software [47] for multilevel data (i.e., clients clustered within staff, clustered within organizations). Multilevel regression analyses of primary and secondary outcomes will be adjusted for baseline dependent measure of interest, age, race, gender, gender identity, sexual orientation, marital status, education level, engagement in HIV medical care during the past 12 months, prescription of ART during the past 12 months, MIBI integrity, and study cohort. Additionally, we will examine the extent to which the relationship between client-level condition assignment and each client-level outcome differs by organization-level condition. Reporting of results will include the coefficient, standard error, corresponding 95% confidence interval, *p* value, and effect size. The Bonferroni method [48] will be used to adjust the overall level of significance for secondary outcomes.

### Monitoring

#### Data monitoring

In addition to being conducted under the auspices of RTI’s IRB, an independent Data and Safety Monitoring Board (DSMB) (see Additional file 2) is used to help with data monitoring. The principal investigator, however, assumes ultimate responsibility for the project’s data and safety monitoring.

#### Harms

At the follow-up assessment, participants are asked to report new adverse symptoms (i.e., unfavorable medical occurrences, symptoms, or diseases), with any adverse event being reported to the principal investigator within 24 h. Adverse events will be reported to the IRB within 2 weeks, while serious adverse events will be reported within 1 week. IRB actions (e.g., approvals, violations) will be reported to the project’s funder in annual progress reports. All adverse events will be reported to the DSMB as part of the annual DSMB report.

#### Auditing

Throughout the participant recruitment period, a project coordinator conducts a weekly check-in with each participating ASO’s trained safety person. Additionally, throughout the follow-up period, a project coordinator conducts a weekly check-in with the follow-up supervisor. Given the number of ASOs, the check-in process is initiated via email, with telephone follow-ups conducted as necessary.

### Ethics and dissemination

#### Research ethics approval

The current study and its full study protocol have been reviewed and approved by RTI’s IRB (Federalwide Assurance No. 3331). As of this writing, the expiration date of the IRB approval is January 7, 2018.

#### Protocol amendments

Any modification to the protocol that may affect the conduct of the study, potential benefit of the participants, or participant safety requires a formal amendment. Such amendments are submitted to RTI’s IRB for review and approval. All protocol amendments are communicated to the DSMB as part of the annual report.

#### Consent

All participants must provide written consent to participate in the project. As described previously, upon completion of the substance use screener, staff use a standardized script to introduce the project to potential participants. Eligible individuals that express interest are read the informed consent, provided a copy for their own records, and given an opportunity to ask questions. Individuals desiring to participate complete the assurance of consent form, which documents (1) that the participant has read the informed consent, (2) that the participant has had the opportunity to ask questions, (3) that the study has been explained to their satisfaction, (4) that the participant has freely decided to participate, (5) that the participant is aware that they may choose to not participate or to withdraw from this study at any time without penalty or loss of benefits to which they are otherwise entitled, (6) the participant’s agreement to participate in the study, (7) the participant’s agreement to the use and disclosure of their information for research study purposes, (8) the participant’s printed name, (9) the participant’s signature, and (10) the date on which the participant provided consent. See Additional file 3 for the project’s introduction script, informed consent, and assurance of consent.

#### Confidentiality

As stated in the informed consent, information provided as part of the study is kept confidential and not shared with anyone outside of the study. The only exception is if participants plan to harm themselves or another specific person. Efforts to protect participant confidentiality were numerous and included (1) assignment of a unique study number only accessible to the ASO study staff and a limited number of RTI study staff; (2) secure storage (e.g., locked file cabinet located in secure building, folder located on password-protected servers located in secure building) of study documents (paper or electronic) that contain both the participant name and study number; (3) not including identifying participant information when study results are presented at meetings or published in journals; and (4) destroying all documents containing identifying information within 90 days of project completion with the exception of the project’s assurance of consent, which must be stored for at least 3 years after study completion.

#### Declaration of interests

There are no competing interests or conflicts of interest to be declared.

#### Access to data

During the active data collection period, data access is restricted to the data coordinator, statistician, and statistical programmer. Upon completion of data collection, full data access will be given to the principal investigator, statistician, and statistical programmer. Upon completion of the project, a public access dataset will be created and made available to the principal investigator upon request.

#### Ancillary and post-trial care

As a strategy to prevent treatment contamination (i.e., participants randomized to the UC condition receiving the MIBI), the importance of strict adherence to protocol during the trial is discussed repeatedly with participating staff. As part of these discussions, it is emphasized that upon completion of participation in the trial, ASOs and their staff are not only allowed, but strongly encouraged, to sustain implementation of the MIBI.

#### Dissemination policy

Irrespective of the magnitude or direction of effect, we will disseminate study findings. Dissemination efforts will include presentations at professional scientific conferences and publication in peer-reviewed journals with the highest impact factor possible. Additionally, we will seek to ensure the project’s publications are open access (i.e., available online to readers without financial, legal, or technical barriers beyond those inseparable from gaining access to the internet).

## Discussion

January 1, 2017, marked the halfway point for the 5-year SAT2HIV Project, a large-scale NIDA-funded type 2 effectiveness-implementation hybrid trial that serves as the parent project for the MIBI Experiment. In this article, the study protocol for the MIBI Experiment, a multisite randomized controlled trial on the effectiveness of MIBI for substance use as an adjunct to usual care in community-based ASOs (Aim 1 of the parent SAT2HIV Project), has been described in accordance with the SPIRIT guidelines [13, 14]. Below, we highlight and discuss trial-relevant events (both anticipated and unanticipated) that have occurred to date, trial-relevant events that remain to be completed, key strengths and weaknesses of the trial, and anticipated impacts of the trial.

### Trial-relevant events that have occurred to date

Table 2 summarizes several anticipated and unanticipated discrete events that have occurred thus far and are helpful in illustrating the MIBI Experiment’s progression and changing outer context. Although not shown in Table 2, important non-discrete trial-relevant events have occurred as well. For example, consistent with our team’s prior research on staff turnover [49–52], some turnover of BI Staff has occurred. This is unfortunate given that extensive resources (both time and financial) are required for BI Staff to demonstrate and maintain MIBI integrity using the ATTC training methods incorporated into this study [53, 54]. To minimize against the negative impact of BI Staff turnover, two BI staff from each ASO were trained in the MIBI. Ideally, more than two BI Staff would have been trained as a further protective measure, but we elected to forego this added protection given the additional resources it would have required and our need to maximize the number of participating ASOs, which is important to maximize statistical power for the SAT2HIV Project’s ISF Experiment (see Fig. 1).

**Table 2:** Key project-relevant events completed to date

Calendar year	Calendar month	Project year	Project month	Key project-relevant events
2014	July	YEAR 1	MONTH 1	The targeted number of participating organizations and client participants was reduced because the grant received a $565,695 reduction in its total budget
	August		MONTH 2	
	September		MONTH 3	
	October		MONTH 4	The Joint United Nations Programme on HIV/AIDS (UNAIDS) released its 90-90-90 treatment targets to help end the AIDS epidemic
	November		MONTH 5	The principal investigator (Dr. Garner) of the grant moved from Chestnut Health Systems to RTI International The grant was relinquished back to the National Institute on Drug Abuse (NIDA)
	December		MONTH 6	
2015	January		MONTH 7	
	February		MONTH 8	The grant award, minus the costs incurred during the first 5 months of the grant, was awarded to RTI International with Dr. Garner again as the principal investigator
	March		MONTH 9	The preparation process for the SAT2HIV Project’s first cohort of AIDS service organizations was initiated
	April		MONTH 10	
	May		MONTH 11	
	June		MONTH 12	The MIBI Experiment preparation process for the SAT2HIV Project’s first cohort of ASOs was completed
	July	YEAR 2	MONTH 13	The MIBI Experiment with the SAT2HIV Project’s first cohort was initiated The updated United States National HIV/AIDS Strategy was released
	August		MONTH 14	
	September		MONTH 15	
	October		MONTH 16	
	November		MONTH 17	
	December		MONTH 18	
2016	January		MONTH 19	The MIBI Experiment with the SAT2HIV Project’s first cohort was completed
	February		MONTH 20	
	March		MONTH 21	The MIBI Experiment preparation process for the SAT2HIV Project’s second cohort of ASOs was initiated
	April		MONTH 22	
	May		MONTH 23	
	June		MONTH 24	The preparation process for the SAT2HIV Project’s second cohort of ASOs was completed
	July	YEAR 3	MONTH 25	The MIBI Experiment with the SAT2HIV Project’s second cohort was initiated
	August		MONTH 26	
	September		MONTH 27	
	October		MONTH 28	
	November		MONTH 29	
	December		MONTH 30	The MIBI Experiment with the SAT2HIV Project’s second cohort was completed

### Trial-relevant events that remain to be completed

The preparation process for the third cohort began March 2017 and will be completed at the end of June 2017, with the effectiveness trial anticipated to begin July 2017 and be completed at the end of December 2017. Upon completion of the final participant follow-up assessments, our research team will initiate the data cleaning and analysis process and the product development and dissemination process.

### Key strengths and limitations of the MIBI Experiment

This SAT2HIV Project’s MIBI Experiment has several noteworthy strengths and limitations. Key weaknesses include (1) the participant sample being limited only to individuals 18 years of age or older who speak English and self-report recent (past 28 days) substance use with indication of a substance use disorder (during the past year) at or above the mild criteria [55], (2) reliance solely on self-reported primary substance use at follow-up as the primary outcome, and (3) a 4–week post-randomization follow-up period. Nonetheless, these limitations are outweighed by the project’s many strengths.

Key strengths include (1) the randomized controlled trial design; (2) conducting the trial in ASOs; (3) the large number of ASOs; (4) the focus on alcohol and other substance use as opposed to a single substance type; (5) the clinical intervention (i.e., MIBI) being tested as an adjunct to usual care rather than as an alternative to usual care (i.e., usual care only vs. brief intervention only); (6) the high degree of internal validity (e.g., blinding follow-up interviewers to condition assignment, extensive quality assurance procedures); and (7) the high degree of external validity (e.g., minimal exclusion criteria, high level of geographic representativeness of ASOs within the United States, delivery of MIBI by ASO case managers).

### Potential impacts of the MIBI Experiment

**Fig. 4:**
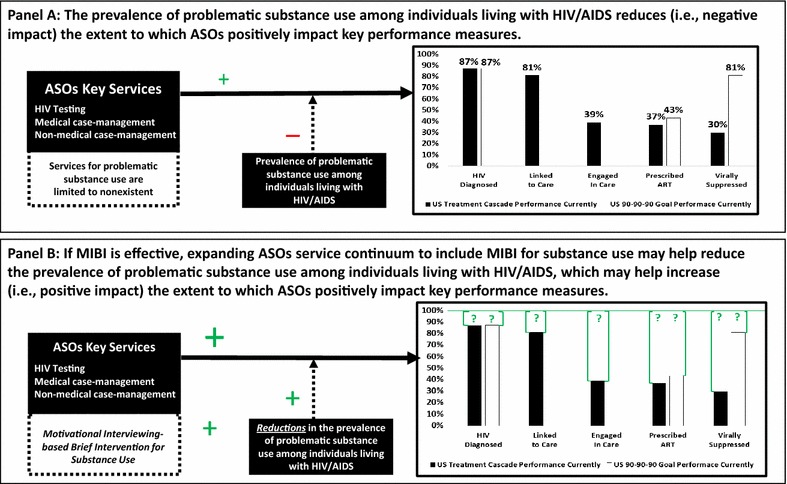
Potential impacts of the SAT2HIV Project’s MIBI Experiment

Panel A of Fig. 4 illustrates the present U.S. performance regarding the UNAIDS 90-90-90 targets [56] and the HIV Care Continuum performance measures [57, 58], as well as that problematic substance use among individuals living with HIV/AIDS reduces the extent to which ASOs and their key services are able to positively impact these key performance measures. Panel B of Fig. 4 illustrates the potential impact of providing support for MIBI as an effective adjunct to usual care within ASOs. More specifically, if MIBI is found to be effective, expanding ASOs’ service continuum to include MIBI for substance use has the potential to help reduce the prevalence of problematic substance use among individuals living with HIV/AIDS, which in turn may increase the extent to which ASOs positively impact key performance measures, such as being linked to care, being engaged in care, being prescribed ART, and achieving viral suppression. However, as indicated by the question marks above each performance measure, future research will be needed to measure changes over time in these performance measures.

The anticipated positive impacts that may ideally stem from reducing problematic substance use among individuals living with HIV/AIDS are posited to stem from increasing ASOs’ ability to help individuals engage in HIV care, which is the most significant break point along the U.S. HIV Care Continuum [59] and has been found to be negatively impacted by substance use [60, 61].

## Conclusion

Comorbid HIV/AIDS and substance use is an issue of great public health relevance given that substance use among people living with HIV/AIDS is associated with several issues including increased psychiatric problems [62], poorer HIV viral suppression [31–33], poorer HIV medication adherence [27–30], and increased likelihood of engaging in risk behaviors that result in infection transmission to others [63]. Thus, with an estimated 50% of adults receiving HIV care reporting substance use in the past 12 months [64], there is a major need to address substance use among people living with HIV/AIDS. The SAT2HIV Project’s MIBI Experiment represents the largest randomized controlled trial to date focused on identifying the best methods to improve ASOs’ ability to address comorbid HIV/AIDS and substance use. Should the effectiveness of MIBI as an adjunct to ASOs’ usual care be supported, our team will seek to support MIBI dissemination, implementation, and sustainment in as many ASOs as possible. Importantly, such efforts will be informed by the SAT2HIV Project’s ISF Experiment (see Fig. 1), which, as previously noted, is testing the effectiveness of a multifaceted implementation strategy as an adjunct to the ATTCs’ current state-of-the-art training model. Consistent with the intent of effectiveness-implementation hybrid designs, our hope is that our design for the parent SAT2HIV Project (i.e., a type 2 effectiveness-implementation hybrid trial) will minimize the research-to-practice lag that has been found to plague numerous areas of health [65–69].

## Additional files

**Additional file 1.** Standard protocol items: recommendations for interventional trials (SPIRIT) checklist.

**Additional file 2.** Data and safety monitoring board (DSMB).

**Additional file 3.** Project's introduction script, informed consent, and assurance of consent.

## Abbreviations

AIDS: acquired immune deficiency syndrome
ASO: AIDS Service Organization
ATTC: Addiction Technology Transfer Center
ART: antiretroviral therapy
BI: brief intervention
DSMB: Data and Safety Monitoring Board
FIPS: Federal Information Processing Standards
HIV: human immunodeficiency virus
ISF: Implementation and Sustainment Facilitation
IRB: Institutional Review Board
MIBI: Motivational Interviewing-Based Brief Intervention
NIDA: National Institute on Drug Abuse
SAT2HIV: Substance Abuse Treatment to HIV
SPIRIT: Standard Protocol Items: Recommendations for Interventional Trials
UNAIDS: Joint United Nations Programme on HIV/AIDS
UC: usual care
