# Correction to: Testing the effectiveness of a motivational interviewing-based brief intervention for substance use as an adjunct to usual care in community-based AIDS service organizations: study protocol for a multisite randomized controlled trial

**DOI:** 10.1186/s13722-018-0107-3

**Published:** 2018-02-21

**Authors:** Bryan R. Garner, Heather J. Gotham, Stephen J. Tueller, Elizabeth L. Ball, David Kaiser, Patricia Stilen, Kathryn Speck, Denna Vandersloot, Traci R. Rieckmann, Michael Chaple, Erika G. Martin, Steve Martino

**Affiliations:** 1 RTI International, P. O. Box 12194, 3040 E. Cornwallis Rd., Research Triangle Park, NC 27709-2194 USA; 2 School of Nursing and Health Studies, University of Missouri-Kansas City, 2464 Charlotte St, Kansas City, MO 64108 USA; 3 University of Nebraska Public Policy Center, 215 Centennial Mall South, Suite 401, Lincoln, NE 68588 USA; 4 Vandersloot Training and Consulting, 11845 NW Stone Mt. Lane, #108, Portland, OR 97229 USA; 5 Public Health and Preventive Medicine, Oregon Health and Science University, 3181 SW Sam Jackson Park Rd. CB669, Portland, OR 97239 USA; 6 National Development and Research Institutes, Inc, 71 West 23rd Street, New York, NY 10010 USA; 7 Department of Public Administration and Policy, Rockefeller College of Public Affairs and Policy, University at Albany, 1400 Washington Avenue, Milne 300E, Albany, NY 12222 USA; 8 Rockefeller Institute of Government, State University of New York, 1400 Washington Avenue, Milne 300E, Albany, NY 12222 USA; 9 Department of Psychiatry, VA Connecticut Healthcare System, Yale University, 950 Campbell Avenue (116B), West Haven, CT 06516 USA

## Correction to: Addict Sci Clin Pract (2017) 12:31 10.1186/s13722-017-0095-8

Following the publication of the original article [1], it was brought to our attention that the abstract contained a minor, yet important error. The last sentence of the abstract’s background section noted: “The current paper describes the study protocol for the ISF Experiment.” However, this sentence should have read: “The current paper describes the study protocol for the MIBI Experiment.” In addition to noting this change as part of the current correction, the original article has been updated to include the correct sentence.

## References

[1] Garner BR, Gotham HJ, Tueller SJ, Ball EL, Kaiser D, Stilen P, Speck K, Vandersloot D, Rieckmann TR, Chaple M, Martin EG (2017). Testing the effectiveness of a motivational interviewing-based brief intervention for substance use as an adjunct to usual care in community-based AIDS service organizations: study protocol for a multisite randomized controlled trial. Addict Sci Clin Pract.

