# Supplementary material for: Testing the effectiveness of a motivational interviewing-based brief intervention for substance use as an adjunct to usual care in community-based AIDS service organizations: study protocol for a multisite randomized controlled trial
Source: Addict Sci Clin Pract. 2017 Nov 17;12:31. doi: 10.1186/s13722-017-0095-8 (PMC5693500; doi:10.1186/s13722-017-0095-8)
Supplement: Supplementary file 2 — Additional file 2. Data and safety monitoring board (DSMB). [file 13722_2017_95_MOESM2_ESM.pdf]

DATA & SAFETY MONITORING BOARD

The Substance Abuse Treatment to HIV care (SAT2HIV) Project includes a Phase III clinical trial focused on testing the effectiveness of a single 20-30 minute motivational interviewing-based brief intervention for substance use within AIDS Service Organizations. In addition to being conducted under the auspices of RTI International's Institutional Review Board (IRB), this project will be conducted with assistance from an independent Data & Safety Monitoring Board (DSMB). Below is the list of the DSMB members.

Sonia Napravnik, PhD (DSMB Chairperson)
University of North Carolina at Chapel Hill
130 Mason Farm Rd, 2101 Bioinformatics Bldg
Chapel Hill, NC 27599-7215
Phone: 919-966-3875
Email: *napravs@med.unc.edu*

Conflict(s) of Interest: NONE

Carol Golin, MD (DSMB member)
University of North Carolina at Chapel Hill
310 Rosenau, Campus Box 7440
Chapel Hill, NC 27599-7440
Phone: 919-966-7939
Email: *carol_golin@med.unc.edu*

Conflict(s) of Interest: NONE

Michael Hudgens, PhD (DSMB member)
University of North Carolina at Chapel Hill
3107-E McGavran-Greenberg
Chapel Hill, NC 27599-7440
Phone: 919-966-7253
Email: *mhudgens@bios.unc.edu*

Conflict(s) of Interest: NONE
