# Supplementary material for: Testing the effectiveness of a motivational interviewing-based brief intervention for substance use as an adjunct to usual care in community-based AIDS service organizations: study protocol for a multisite randomized controlled trial
Source: Addict Sci Clin Pract. 2017 Nov 17;12:31. doi: 10.1186/s13722-017-0095-8 (PMC5693500; doi:10.1186/s13722-017-0095-8)
Supplement: Supplementary file 3 — Additional file 3. Project's introduction script, informed consent, and assurance of consent. [file 13722_2017_95_MOESM3_ESM.pdf]

PROJECT INTRODUCTION SCRIPT

Based on the information you provided as part of the project's screener form, you appear eligible for a project our HIV/AIDS organizations is participating in.

This project is funded by the National Institute on Drug Abuse and is focused on learning to what extent a one-time 20-30 minute brief intervention is helpful in reducing alcohol and/or substance use among individuals living with HIV/AIDS.

Study participation is voluntary and not participating will not cause you to lose any benefits to which you are otherwise entitled.

Study participation includes the completion of an interview at the beginning of the study, as well as 4-weeks later.

Because we understand your time is valuable, each time an interview is completed, a $20 gift card will be provided as compensation.

Does this project sound like something you would like to learn more about?

Client Consent to Participate in Research

Title of Research: Project Spark

Introduction

Because of the information you provided about your use of substances, you are being asked to participate in a study. Before you decide if you want to take part in this study, you need to read this Informed Consent form so that you understand what the study is about and what you will be asked to do. This form also tells you who can be in the study, the risks and benefits of the study, how your information will be protected, and who you can call if you have questions. Please ask to have anything you don't understand explained before you make your decision.

Purpose

This study is called Project Spark. It is paid for by the National Institute on Drug Abuse. The study is being conducted by RTI International, a research organization located in North Carolina. The purpose of this study is to see if a brief intervention can help reduce alcohol or substance use among people living with HIV/AIDS. You are one of about 72 clients within this AIDS Service Organization (ASO) who are being asked to participate in this study, as well as one of approximately 2,600 clients across the United States that are being asked to participate in this national study.

Procedures

Alternative Care Options.

Please note that you may choose to continue with the usual care provided by this ASO and not be in the study.

If you agree to participate, you will be asked to consent to the following:

1. Consent to be randomly assigned to study group.

The way we learn if this brief intervention works is to provide the brief intervention to half of study participants, but not the other half. If you agree to participate, you are agreeing to be put in one of the two groups listed below. Because group assignment is random, you will not be able to choose which group you are in.

Group 1: Usual care.

Group 2: Usual care AND a 20-30 minute brief intervention session.

This brief intervention will be provided by a trained staff person from this ASO. They will seek to understand how you view your alcohol or substance use. They also will help you explore motivations you may have for wanting to reduce your alcohol or substance use.

2. Consent to have brief intervention session audio recorded.

In order to see how well staff delivering the brief intervention are doing, all brief intervention sessions will be recorded. If you agree to participate and are assigned to Group 2, you are agreeing to have your brief intervention session recorded for research purposes. Recordings will not include your name. They will only be listened to by study staff for the purposes of seeing how well the brief intervention is working. If you have something private to say, the recorder can be paused at any time. If any information, such as your name or where you live, is included on the recording by accident this information will be removed from the recording by a member of the study team. All recordings will be kept by the study team and may be used for future research purposes.

3. Consent to complete an initial interview and a follow-up interview.

Regardless of which of group you are assigned, you are being asked to tell us about your background, your alcohol and/or other substance use, any recent alcohol or substance use treatment you may have received or are receiving, and your use of any HIV/AIDS medications. If you agree to be part of this study, you will take part in two interviews that last approximately 20-minutes each. The first interview will be done in-person by staff from this ASO. Information collected as part of the first interview may be used by the ASO to help plan your care. The second interview will be over the phone by study staff. Information collected as part of this second interview will only be used for the study. It will NOT be shared with anyone at this ASO or outside of the study team.

4. Consent to provide contact information.

You will need to be contacted in order to complete the follow-up interview. If you agree to participate, you are agreeing to provide contact information (e.g., mailing address, phone number, and email address) for both you and at least one other individual who you believe will help us get in contact with you to complete the follow-up telephone interview.

Study Duration

Your involvement in the study will last for approximately 1 month.

Possible Risks or Discomforts

There are no physical risks involved in this study. Although unlikely, it is possible that one or more questions may make you uncomfortable. You can refuse to answer any question or you may take a break at any time during the survey. There is also a potential risk of loss of confidentiality. Please note that every effort will be made to protect your information, but this cannot be guaranteed. In addition to the risks and discomforts listed here, there may be uncommon or previously unknown risks. You should report any problems to Dr. Bryan Garner.

Benefits

Your Benefits

There are no direct benefits to you for participating in this study.

Benefits for Other People

If this brief intervention works, it may help other people living with HIV/AIDS and who use alcohol and/or substances.

Payment for Participation

You will receive a **$20** gift card for completing the first interview and a **$20** gift card for completing the second interview. This is a **$40 total value**.

Confidentiality

The information you provide as part of this study is confidential and will not be shared with anyone outside of the study. The only exception is if you tell us that you plan to harm yourself or plan to harm another specific person. Many efforts have been taken to protect your information. You will be assigned a study number. Only staff from this ASO and other study staff will have access to your name and your study number. Any document (paper or electronic) that contains both your name and study number will be securely stored (e.g., locked file cabinet located in secure building, folder located on one of RTI's password protected servers located in secure building). If the results of this study are presented at meetings or published in journals, no information will be included that could identify you or your answers. All documents that contain identifying information will be destroyed within 90 days of project completion. The only exception is the project's Assurance of Consent Form that must be stored for at least three years after study completion.

Future Contacts

No future contacts are planned.

Your Rights

Your decision to take part in this study is completely voluntary. You can refuse any part of the study and you can stop participating at any time. You can refuse to answer any questions. If you decide to participate and later change your mind, you will not be contacted again or asked for further information. If you decide not to participate in the study, or you withdraw later, you will not lose any benefits to which you are otherwise entitled.

Your Questions

If you have any questions about the study, you may contact Dr. Bryan Garner by calling him at (919) 597-5159 or emailing him at bgarner@rti.org. If you have any questions about your rights as a study participant, you may call RTI's Office of Research Protection at 1-866-214-2043.

PROJECT SPARK

PROJECT ASSURANCE OF CLIENT CONSENT

I have read the above informed consent for Project Spark. I have had the opportunity to ask questions about the study and the study has now been explained to my satisfaction. I have freely decided to participate in this study. I am aware that I may choose not to participate or to withdraw from this study at any time without penalty or loss of benefits to which I am otherwise entitled.

Please indicate your decision regarding participation Project Spark by completing the appropriate box below. Thank you!

AGREE to Participate	DO NOT AGREE to Participate
I (see name below) hereby agree to participate in this study and to the use and disclosure of my information for research study purposes.	I (see name below) hereby DO NOT AGREE to participate in this study.<br>My reason(s) for not participating is/are: (please note below).<br>____________________<br>____________________
____________________<br>Name (please print)	____________________<br>Name (please print)
____________________<br>Signature	____________________<br>Signature
____________________<br>Date	____________________<br>Date<br>We will keep your information for 90 days
